# Relationship between hand osteoarthritis severity and serum resistin levels in women with metabolic syndrome: a case-control study

**DOI:** 10.55730/1300-0144.5964

**Published:** 2024-10-07

**Authors:** Ekin Başak DOĞANCI, Berna İmge AYDOĞAN, Nilgün BAŞKAL, Safiye TUNCER

**Affiliations:** 1 Department of Rheumatology, Sivas Numune Hospital, Sivas, Turkiye; 2 Department of Endocrinology and Metabolic Diseases, Güven Hospital, Ankara, Turkiye; 3 Department of Endocrinology and Metabolic Diseases, Faculty of Medicine, Ankara University, Ankara, Turkiye; 4 Department of Physical Medicine and Rehabilitation, Faculty of Medicine, Ankara University, Ankara, Turkiye

**Keywords:** Osteoarthritis, hand, metabolic syndrome, resistin

## Abstract

**Background/aim:**

The aim of this study was to determine the relationship between clinical and radiological hand osteoarthritis (OA) severity and serum resistin levels in women diagnosed with metabolic syndrome (MetS).

**Materials and methods:**

This research was designed as a case-control study. A total of 87 women, aged 50–65 years, were evaluated between January to March 2014, comprising 29 patients diagnosed with MetS and hand OA (MetS (+), hand OA (+); group 1), 29 patients without MetS but with hand OA (MetS (−), hand OA (+); group 2), and 29 healthy controls (MetS (−), hand OA (−); group 3). The diagnostic criteria of the American College of Rheumatology (ACR) for hand OA and National Cholesterol Education Program Adult Treatment Panel III (NCEP-ATP III) for MetS were used. The presence and severity of pain were evaluated according to the Likert scale, function by measuring grip and pinch strength, the presence of disability by Duruöz hand score (DHS), and structural damage by the Osteoarthritis Research Society International (OARSI) atlas classification.

**Results:**

There were no significant differences between the three groups in terms of the presence and severity of pain, functional assessment parameters, disability scores, and serum resistin levels. Structural damage was more severe in group 2. No significant association was found between the serum resistin levels and pain severity, functional assessment parameters, disability scores, hand OA findings (total number of tender joints, the total number of nodules, etc.), and structural damage.

**Conclusion:**

MetS did not contribute to clinical and radiological hand OA severity. Serum resistin levels were not higher in patients with MetS and had no effect on hand OA severity.

## 1. Introduction

Osteoarthritis (OA) is a progressive, degenerative joint failure that increases with age. It is known that obesity increases the risk of OA [1]. Obesity-related OA is attributed to the increase in loading on weight-bearing joints, while it is attributed to the presence of metabolic factors in nonweight-bearing joints such as hand joints.

In obesity, the white adipose tissue that accumulates around the organs acts as a metabolically active, endocrine organ by secreting adipocytokines such as leptin, adiponectin, and resistin, and inflammatory cytokines. Resistin acts as a proinflammatory mediator rather than increasing insulin resistance and it is probably associated with subclinical inflammation [2]. In the pathogenesis of OA in nonweight-bearing joints, these adipocytokines are thought to directly have a role, independent of the mechanical effect. Recent studies have revealed that in the pathogenesis of OA, obesity does not play a role alone. It has been reported that the incidence and prevalence of OA are increased in obese individuals with cardiometabolic disorders compared to obese individuals without these disorders. In light of these findings, OA has begun to be accepted as the fifth component of metabolic syndrome (MetS), and as a new OA phenotype, OA-associated MetS (MetS-OA) has recently begun to be used as terminology [2].

The aim of this study was to determine the relationship between clinical and radiological hand OA severity and serum resistin levels in individuals diagnosed with MetS and also investigate the contribution of serum resistin levels to clinical and radiological hand OA severity.

## 2. Materials and methods

This research was designed as a case-control study. A total of 87 women, aged 50–65 years, who were evaluated at our outpatient clinic between January to March 2014 were included in the study. Patients diagnosed with MetS according to National Cholesterol Education Program Adult Treatment Panel III (NCEP-ATP III) [3] criteria with hand pain by the same endocrinologist were referred to our outpatient clinic. Group 1 included 29 women diagnosed with MetS as well as hand OA according to the American College of Rheumatology (ACR) [4] diagnostic criteria [MetS (+), hand OA (+)]. Group 2 included 29 women with a diagnosis of hand OA but without MetS according to the ACR diagnostic criteria [MetS (−), hand OA (+)]. Group 3 (control group) comprised 29 healthy volunteers [MetS (−), hand OA (−)]. The diagnosis of MetS was made at the endocrine outpatient clinic, according to the NCEP-ATP III diagnostic criteria. Informed consent was obtained from each participant and the study was conducted in line with the ethical guidelines for human experimentation and the latest version of the Declaration of Helsinki. This study was approved by the Ethics Committee of Ankara University Faculty of Medicine clinical research, Ankara, Türkiye (date: February 10, 2014) (decision number: 03-86-14). Patients who had mental disorders making it difficult for them to understand tests or with neurological defects that may affect hand skills, chronic widespread pain syndrome, radial, ulnar, or median nerve entrapments, cervical radiculopathy, a history of hand surgery, a history of hand trauma resulting in the deformity in the affected joints, inflammatory rheumatological diseases with hand joints involvement, those who had an injection or physical therapy modality for the hand joints in the last 3 months were excluded. Moreover, those with malignancy, pregnancy and lactation, liver and kidney failure, active inflammatory or infectious systemic disease, and those using opioids for pain were excluded from the study. The demographic data, personal and osteoarthritic family history, risk factors for OA, and physical examination findings of the participants were recorded.

### 2.1. Measurements

The presence and severity of pain in the hand joints were determined according to the Likert scale (0–5) [5]. High scores indicated that the pain was severe.

The Jamar hand dynamometer and Jamar pinchmeter were used for functional evaluation. Grip strength was measured with the Jamar hand dynamometer [6–8]. Lateral finger grip was measured with the Jamar pinchmeter [6–8]. High grip strength indicated relatively good hand function.

Functional disability was measured according to the Duruöz hand scale [9]. High scores indicated a functional disability.

Radiographic evaluation was planned only for the two groups with hand OA. The patients were evaluated by a blinded investigator using posteroanterior hand radiography according to the Osteoarthritis Research Society International (OARSI) atlas classification [10]. High scores indicated severe radiological damage.

Blood was drawn from the antecubital vein of each participant into an ethylenediaminetetraacetic acid tube and after centrifugation, the samples were stored at −80 °C until use. All the samples were examined using a human resistin ELISA kit (Boster Biological Technology Co. Ltd., Pleasanton, CA, USA) and the measured resistin values were recorded. The sensitivity was <3 pg/mL and the assay range was 78–5.000 pg/mL.

### 2.2. Statistical analysis

The data were analyzed using SPSS Statistics for Windows 15 (SPSS Inc., Chicago, IL, USA). Descriptive statistics were expressed as the mean ± standard deviation (SD) for variables with normal distribution, and as the median (min–max) for variables with nonnormal distribution. The nominal variables were expressed as numbers and percentages (%). For the comparison of two groups, the difference in the mean values was analyzed using the t test, and the difference in the median values was analyzed using the Mann–Whitney U test. For the comparison of three groups, the difference in the mean values was investigated using analysis of variance (ANOVA), and the difference in the median values was investigated using the Kruskal–Wallis test. Nominal variables were evaluated using the Pearson’s chi-squared or Fisher’s exact test. While investigating the relationship between continuous variables, Spearman’s correlation test was used for nonnormally distributed values, and Pearson’s correlation test was used for values with normal distribution. The number of required patients was determined using power analysis. p < 0.05 was considered as statistically significant.

## 3. Results

There was a statistically significant difference between the three groups [MetS (+), hand OA (+); MetS (−), hand OA (+); MetS (−), hand OA (−)] in terms of the body mass index (BMI), and this difference was caused by the group with MetS (+) and hand OA (+), as shown in Table 1.

Most of the patients in the two groups with hand OA were housewives (p < 0.05), as shown in Table 2.

A statistically significant difference was found between the three groups in terms of the Likert scale and Duruöz hand scale scores, and this difference was due to the MetS (−) and hand OA (−) group (group 3), as shown in Table 3.

Structural damage was statistically significant between groups 1 and 2 (p = 0.004 and p < 0.001, respectively). The mean value in group 2 was higher on both the right (28.3) and the left (29.28) compared to group 1, as shown in Table 4.

A statistically significant difference was found in the hand OA findings of the three groups. When a pairwise comparison was made, there were statistically significant differences between groups 1 and 3, and between groups 2 and 3 (p < 0.05), while no statistically significant difference was found between groups 1 and 2.

Of the three groups, the presence of radial/ulnar deviations was in favor of the control group (p < 0.05).

When the participants were evaluated independently of the presence of MetS, no statistically significant difference was found between serum resistin levels and pain severity, functional evaluation parameters, and hand OA findings, as shown in Table 5.

When the patients with hand OA were evaluated independently of MetS, no association was found between the serum resistin levels and structural damage. However, there was a very strong positive association between the serum resistin levels and first right carpometacarpal (1st R_CMC) joint erosion (p = 0.001, r = 0.534), a moderately strong positive association between second right proximal interphalangeal (2nd R_PIP) joint erosion (p = 0.002, r = 0.401), and a moderately strong positive association between third right proximal interphalangeal (3rd R_PIP) joint erosion (p = 0.002, r = 0.401), as shown in Table 6.

## 4. Discussion

The pathogenesis of OA is multifactorial. Many factors such as biomechanical, genetic, metabolic, and neuroendocrine factors are blamed for this [11]. The primary target in the affected tissue is cartilage. Inflammatory changes were also seen in the synovial tissue and subchondral bone. These inflammatory changes are thought to be related to the increase in inflammatory cytokine and proteolytic enzyme production [12]. Increased body weight is thought to be responsible for cartilage degeneration, especially in weight-bearing joints such as the hips and knees. However, the effect of local or systemic inflammatory metabolic pathways apart from the mechanical load on the pathogenesis of OA in nonweight-bearing joints, such as the hand, has been intensively investigated in recent years [13].

Among the metabolic factors, cytokines and adipocytokines released from adipose tissue are often the suspected cause. In particular, it is thought that white adipose tissue functions as a metabolically active endocrine organ and plays a role in the release of inflammatory cytokines and adipocytokines [14]. Furthermore, it has been suggested that these adipocytokines play a role in the emergence of other components of MetS.

The role of adipocytokines in the pathogenesis of OA has been investigated for a long time. However, their true role in the pathogenesis of OA and its relationship with OA is still unclear [15].

Resistin, one of the adipocytokines, is a cysteine-rich polypeptide hormone released from white adipose tissue, mononuclear cells, bone marrow cells, and macrophages in humans and mice. There are reports that resistin increases insulin resistance in mice. Resistin blood levels vary depending on leptin activity. It was reported that serum resistin levels increase in mouse models in which leptin activity is blocked [16]. In human models, it acts as a proinflammatory mediator rather than increasing insulin resistance and it is probably associated with subclinical inflammation. Therefore, resistin can be detected locally in the inflamed joint synovium in both rheumatoid arthritis and OA [17]. In addition, increased plasma levels of resistin in obesity suggest that it is a potential mediator for obesity and inflammation-related OA [14].

In previous studies [14,16,17], the relationship between obesity, resistin, and OA was investigated. Nevertheless, to the best of our knowledge, the current study is the first to investigate the effect of MetS on hand OA regarding resistin. When the three groups herein were compared in terms of the serum resistin levels, no difference was found (especially between groups 1 and 2). In one study, no difference was found between the erosive and nonerosive hand OA group and the control group in terms of the serum resistin levels, while adiponectin was shown to be higher in the erosive subtype. Moreover, in the same study, no correlation was found between the adiponectin/resistin levels and C-reactive protein or BMI [18]. In another study, the relationship between leptin, adiponectin, and resistin with long-term progression in hand OA was investigated. While no effect was detected for leptin and resistin on the long-term progression of hand OA, the mean adiponectin level was significantly lower in patients with progression [19].

In the present study, a significant difference was only found in the Likert scale and Duruöz hand scale parameters between the three groups, in favor of the control group. However, pain severity, function, and disability parameters were not more severe in women with MetS and hand OA. On the contrary, Marshall et al. [20] reported that dyslipidemia, hypertension, and MetS were more common in patients with erosive hand OA. In a study by Sanchez-Santos et al. [21], a statistically significant relationship was found between radiographic hand OA accompanied by pain in the interphalangeal joints and hypertriglyceridemia, high high-density lipoprotein levels, hypertension, and high fasting glucose levels, with the strongest relationship with the high fasting glucose level [21]. Their results contradict those found in the current study. However, Strand et al. [22] detected no relationship between hand OA and MetS. Moreover, they demonstrated that hypertriglyceridemia had a protective effect. Caspi et al. [23] found no relationship between hand OA and diabetes mellitus, obesity, and hypothyroidism. Therefore, the true relationship between MetS and hand OA remains unclear.

When the structural damage was evaluated using the OARSI classification, the mean right and left total OARSI classification scores were higher in group 2. This result may have been related to the evaluation of structural damage with a method such as direct radiography, or possibly due to the fact that direct radiography is an observer-dependent method. We may consider the fact that more sensitive methods such as ultrasonography or magnetic resonance should be used in the evaluation of structural damage.

When the volunteers in the study were evaluated independently of MetS, there was no relationship between the serum resistin levels and pain severity, functional evaluation parameters, hand OA findings, and structural damage. This finding contradicts a previous study that found a relationship between the presence of subchondral erosion and serum resistin levels in patients with hand OA [24]. However, considering the current results, it can be said that serum resistin levels do not play a role in structural damage in hand OA. The low inflammatory response detected in hand OA may be mediated by other adipocytokines or other proinflammatory cytokines. In the present study, when the relationship between the OARSI subgroups and serum resistin levels was evaluated, a very strong positive association between the serum resistin levels and 1st R_CMC joint erosion, a moderate positive association between 2nd R_PIP joint erosion, and a positive moderate association between 3rd R_PIP joint erosion was found. This result was not only related to resistin but could also be related to hand biomechanics. Since the distal interphalangeal (DIP) joint has the smallest contact area compared to other joints, the average contact pressure is also highest in the DIP joint. This is a risk factor for DIP joint involvement for hand OA [25]. In one study, the contact pressure forces in all three joints of the index finger (metacarpophalangeal (MCP), PIP, and DIP) during isometric hand functions were examined. Accordingly, the MCP and DIP joint pressure forces were higher than the PIP joint pressure forces in activities with a fine grip, such as key holding, end-to-end holding, and pulp holding [26]. Pressure forces in the MCP and PIP joints were higher than the DIP joint pressure forces in activities with coarse grip such as hook grip and spherical grip. Therefore, the type of grip that is frequently used in daily life can determine the presence or frequency of DIP or PIP joint OA. When the thumb was also evaluated, the joint pressure and joint strength on the CMC joint were higher than both the distal thumb joints and the other finger joints, since the mass of muscle that passes the joint, and thus the muscle contraction, increases in the proximal side. Therefore, the CMC joint is also a target for hand OA [25]. Considering these findings, we believe that the abovementioned erosion should be explained not only by the metabolic effect but also by the mechanical effect.

Hence, it was aimed to investigate the effect of MetS on hand OA through resistin in the current study. In addition, although some reports have suggested that hand OA is associated with obesity, and therefore MetS, the effects of obesity and MetS on pain, function, and disability have not been investigated before. In fact, a study in 2011 found no association of resistin and leptin with the long-term progression of hand OA [19]. There have also been some studies [27,28] reporting that there is no relationship between hand OA and MetS. The current study also supported these results.

There were some limitations of this study. First, it was a case-control study with a small sample size. Moreover, as it was not a cohort study, it was difficult to predict disease progression, the structural damage was evaluated only using a less sensitive method, such as direct radiography, the assessment of structural damage by direct radiography was observer-dependent, and the healthy controls were very hands-on and hand strength selection from high professional groups (nurses, physiotherapists). Studies with a larger sample size are still needed to fully elucidate the relationship between serum resistin levels and MetS.

## 5. Conclusion

The pathogenesis of hand OA is multifactorial. In pathogenesis, there is a complex effect of both metabolic and mechanical factors, which play a role together. Herein, no relationship was found between MetS and hand OA or between the serum resistin levels and hand OA. This suggests that biomechanical factors should not be ignored in the pathogenesis of OA in nonweight-bearing joints such as the hand. However, studies with a larger sample size are still needed. Further investigations to prove that the effect of MetS on hand OA is mediated by adipocytokines are warranted.

## Figures and Tables

**Table 1:** Comparison of the groups.

Variables	Group 3 (n = 29)	Group 1 (n = 29)	Group 2 (n = 29)	p-value
	Mean ± SD (min–max)	Mean ± SD (min–max)	Mean ± SD (min–max)	
**Age (year**s**)**	58.21 ± 4.49 (51–65)	58.90 ± 4.31 (51–65)	58.07 ± 4.50 (50–65)	0.749
**Duration of complaint (year**s**)**	-	3.00 (0.58–18)	3.00 (1–10)	0.284
**BMI (kg/m** **^2^** **)**	24.29 ± 1.84 (20.3–28.4)	31.50 ± 4.56 (22.0–41.5)	25.40 ± 2.92 (19.3–31.4)	<0.001

Group 1: MetS (+), hand OA (+), group 2: MetS (−), hand OA (+), and group 3: MetS (−), hand OA (−).

**Table 2:** Demographic and clinical characteristics of the groups.

Variables	Group 3		Group 1		Group 2	

	n	%	n	%	n	%

Profession						
**Housewife**	8	27.6	24	82.8	18	62.1
**Worker**	21	72.4	5	17.2	11	37.9

Education status						
**Literate**	2	6.9	8	27.6	1	3.4
**Illiterate**	27	93.1	21	72.4	28	96.6

Menopause						
**No**	3	10.3	2	6.9	1	3.4
**Yes**	26	89.7	27	93.1	28	96.6

Smoke						
**No**	21	72.4	26	89.7	23	79.3
**Yes**	8	27.6	3	10.3	6	20.7

Dominant hand						
**Right**	28	96.6	28	96.6	29	100
**Left**	1	3.4	1	3.4	0	0

Hand with complaint						
**Right**			17	58.6	18	62.1
**Left**	-	-	9	31.1	3	10.3
**Bilateral**			3	10.3	8	27.6

J**oint laxity**						
**No**	26	89.7	29	100	28	96.6
**Yes**	3	10.3	0	0	1	3.4

OA family history						
**No**	22	75.9	22	75.9	18	62.1
**Yes**	7	24.1	7	24.1	11	37.9

**Table 3:** Relationship between the evaluation scales and the study groups.

Evalution scales	Group 3	Group 1	Group 2	p-value
	Mean ± SD (min–max)	Mean ± SD (min–max)	Mean ± SD (min–max)	
**Serum resistin levels**	2.98 (1.69–5.95)	2.75 (1.67–9.66)	2.87 (1.82–13.08)	0.790
**Likert scale**	0.00 (0–0)	2.00 (1–4)	1.00 (1–3)	<0.001
**Duruöz hand scale**	0.0 (0–4)	6.00 (0–37)	4.00 (0–50)	<0.001
**Right pinch (kg/force)**	6.00 (4.5–9.5)	6.00 (3–9)	6.00 (1–10)	0.370
**Left pinch (kg/force)**	6.00 (3.5–10)	5.50 (2.5–10)	6.00 (1–18)	0.950
**Right grip strenght (kg/force)**	18.28 ± 6.01 (6–32)	17.29 ± 5.62 (8–32)	19.30 ± 8.25 (4–40)	0.524
**Left grip strenght (kg/force)**	17.45 ± 6.28 (8–34)	16.03 ± 6.06 (5–27)	18.34 ± 7.65 (2–36)	0.420

**Table 4:** OARSI classification score structural damage evaluation.

Total OARSI classification scores	Group 1	Group 2	p-value
	Mean ± SD (min–max)	Mean ± SD (min–max)	
**Right total OARSI**	21.45 ± 6.97 (11–36)	28.30 ± 9.47 (13–50)	0.004
**Left total OARSI**	20.69 ± 6.39 (6–35)	29.28 ± 8.22 (13–44)	<0.001

**Table 5:** Relationship between the serum resistin levels and the evaluation scales and hand OA findings.

Evaluation scales		Likert	Duruöz	Right pinch	Left pinch	Left Jamar	Right Jamar
**Serum resistin levels (n = 87)**	r	0.050	0.132	−0.014	−0.089	−0.058	−0.129
	p-value	0.647	0.225	0.896	0.410	0.593	0.232

Hand OA findings		Total number of nodules	Total number of sensitive joints
**Serum resistin levels (n = 87)**	r	0.053	0.054
	p-value	0.625	0.621

**Table 6:** Relationship between the serum resistin levels and the total OARSI classification scores and OARSI classification subgroups.

Total OARSI scores		Right total OARSI scores	Left total OARSI scores
**Serum resistin levels (n = 58)**	r	−0.040	0.071
	p-value	0.767	0.599

OARSI subgroups		1st R_CMC erosion	2nd R_PIP erosion	3rd R_PIP erosion
**Serum resistin levels (n = 58)**	r	0.534	0.401	0.401
	p-value	0.001	0.002	0.002

1st R_CMC: first right carpometacarpal, 2nd R_PIP: second right proximal interphalangeal, and 3rd R_PIP: third right proximal interphalangeal.
